# Supplementary figures and images for: Transitions from hospital to home: A mixed methods study to evaluate pediatric discharges in Uganda
Source: PLOS Glob Public Health. 2023 Sep 13;3(9):e0002173. doi: 10.1371/journal.pgph.0002173 (PMC10499195; doi:10.1371/journal.pgph.0002173)

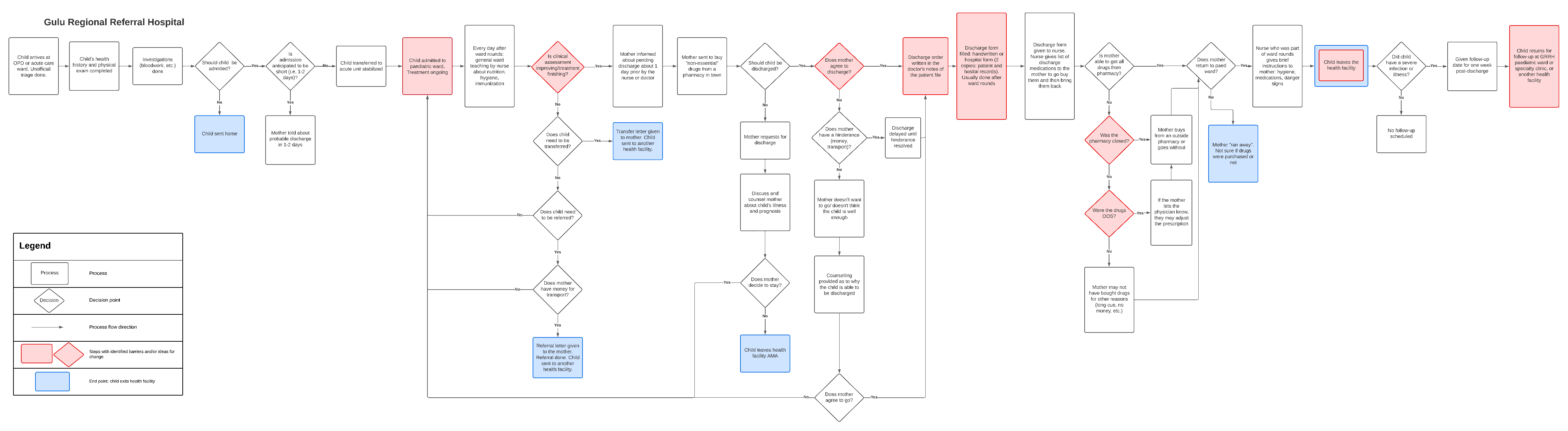

Supplement: S1 Fig — (TIF) [file pgph.0002173.s001.tif]

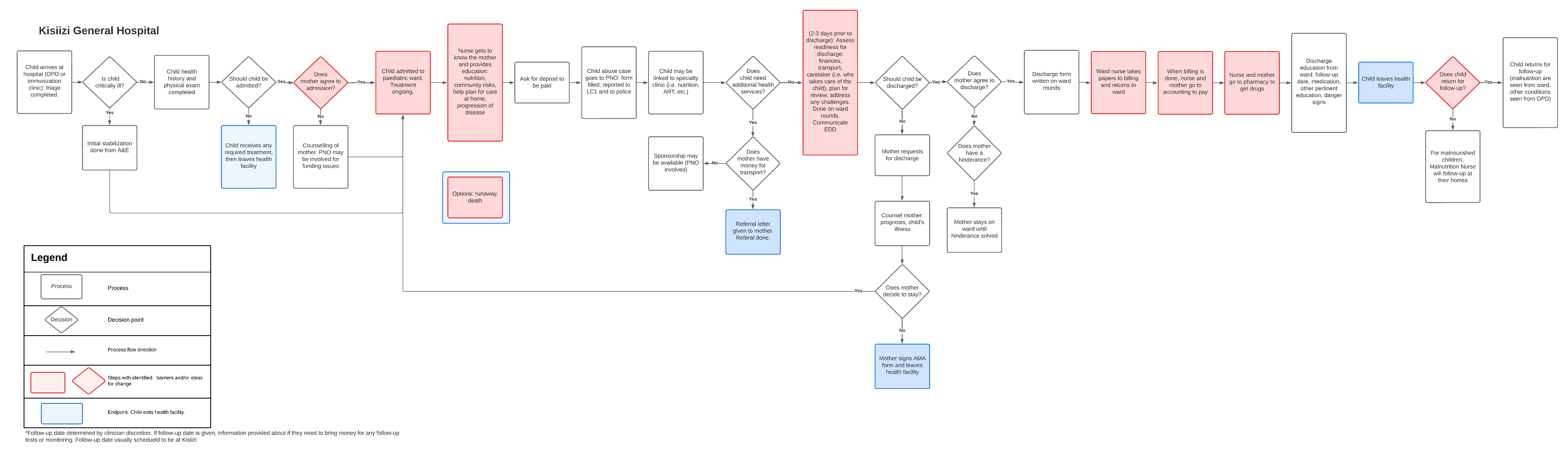

Supplement: S2 Fig — (TIF) [file pgph.0002173.s002.tif]

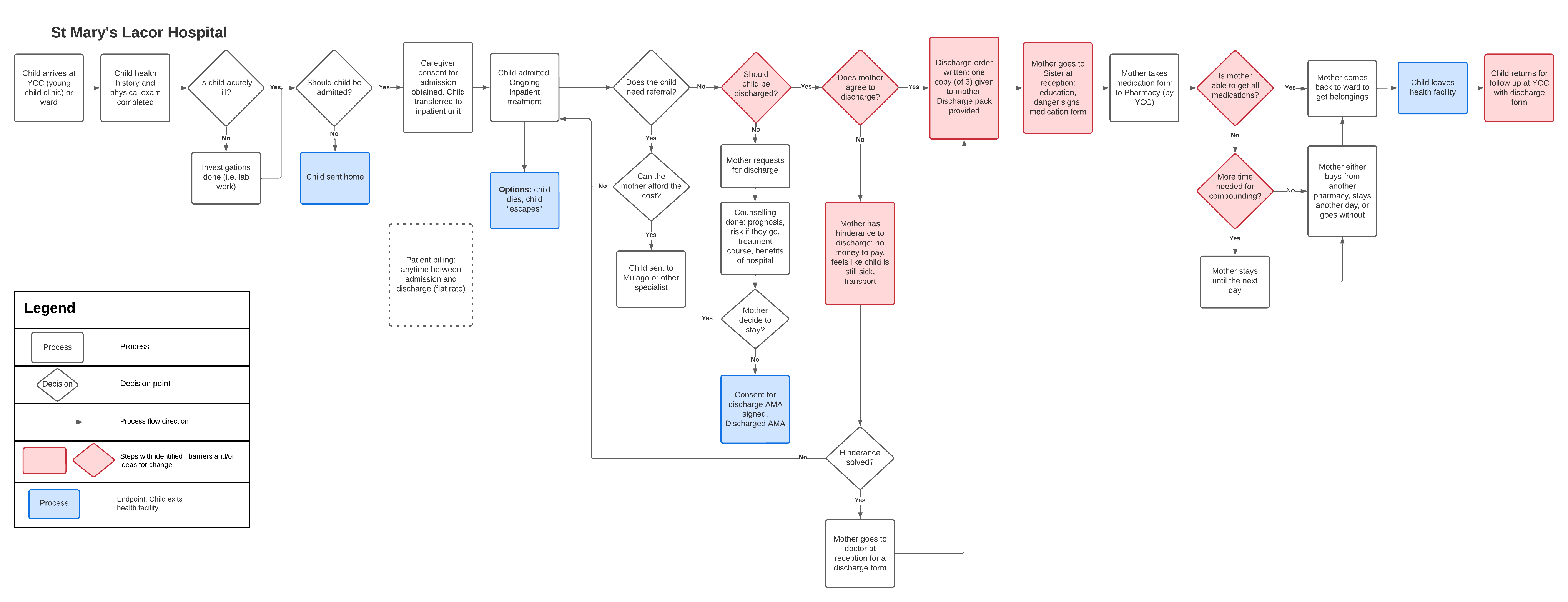

Supplement: S3 Fig — (TIF) [file pgph.0002173.s003.tif]

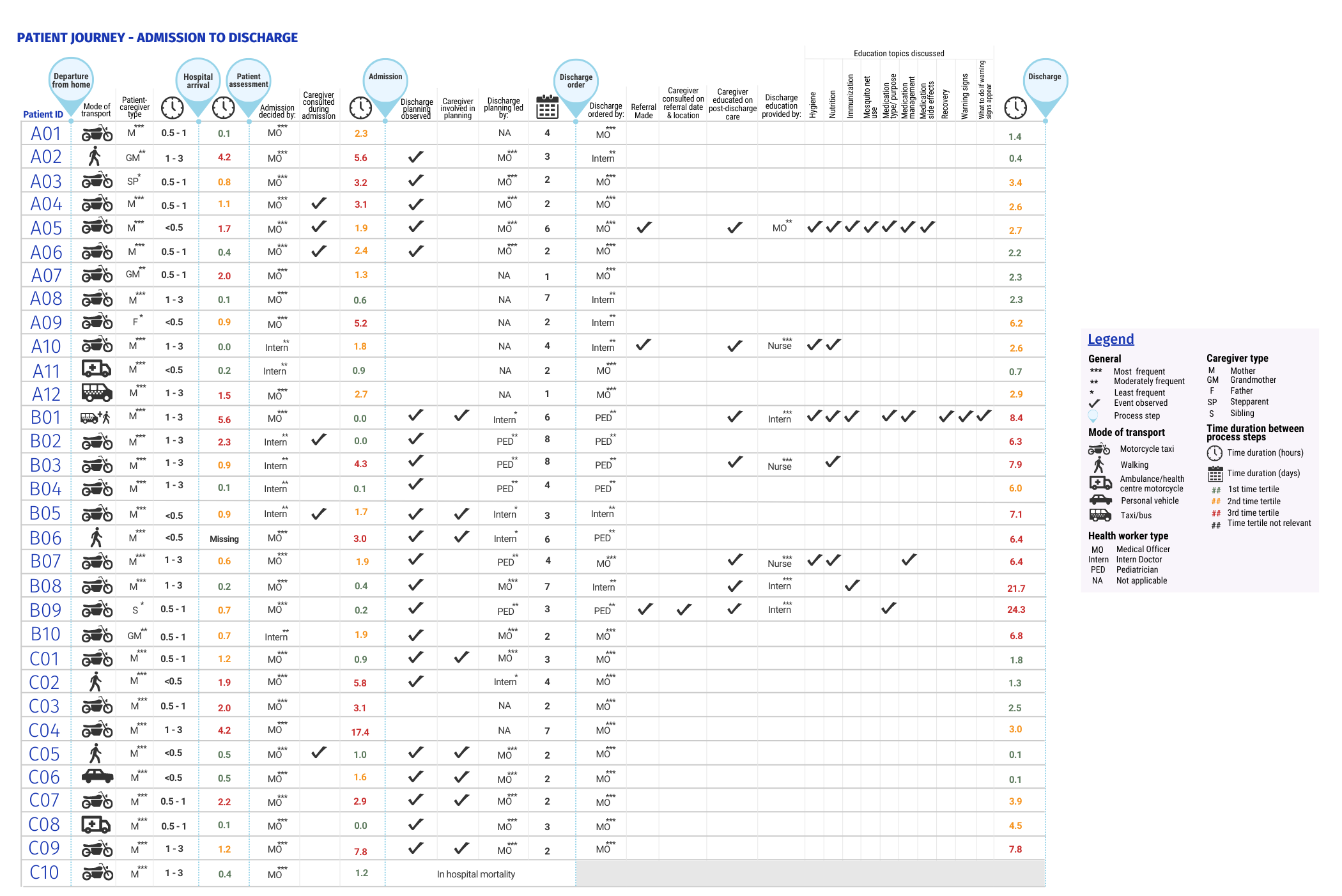

Supplement: S4 Fig — (TIF) [file pgph.0002173.s004.tif]
