# Supplementary material for: Transitions from hospital to home: A mixed methods study to evaluate pediatric discharges in Uganda
Source: PLOS Glob Public Health. 2023 Sep 13;3(9):e0002173. doi: 10.1371/journal.pgph.0002173 (PMC10499195; doi:10.1371/journal.pgph.0002173)
Supplement: S1 Text — (DOCX) [file pgph.0002173.s005.docx]

**Transitions from hospital to home: A mixed methods study to evaluate pediatric discharges in Uganda.**

**Supplementary File 4: Tables**

| **S4 Table A.** **Patient Journey** | |  | | |
| --- | --- | --- | --- | --- |
|  | | **n** | | **%** |
| Travel to facility (N=32) | |  | |  |
| Child brought to hospital by: | |  | |  |
| Biological father | | 1 | | (3.1) |
| Biological mother | | 26 | | (81.3) |
| Grandmother | | 3 | | (9.4) |
| Sibling | | 1 | | (3.1) |
| Stepmother | | 1 | | (3.1) |
| Mode of transportation to hospital | |  | |  |
| Ambulance | | 1 | | (3.1) |
| Health centre motorbike | | 1 | | (3.1) |
| Motorcycle taxi | | 22 | | (68.8) |
| Bus/Taxi | | 1 | | (3.1) |
| By foot | | 5 | | (15.6) |
| Both Taxi and by foot | | 1 | | (3.1) |
| Personal vehicle | | 1 | | (3.1) |
| Travel time duration from home to health facility | |  | |  |
| <0.5 hours | | 8 | | (25.0) |
| 0.5 – < 1 hour | | 11 | | (34.4) |
| 1 hour - <3 hours | | 13 | | (40.6) |
| Arrival to Assessment (N=32) | |  | |  |
| Time duration (hours) between hospital arrival and patient assessment (N=31), Median (IQR) | | 0.8 | | (0.3 – 1.8) |
| Patient referred for current admission | | 8 | | (25.0) |
| Place patient was referred from for current admission (N=8) | |  | | |
| Health centre / clinic | | 6 | | (75.0) |
| Private hospital | | 2 | | (25.0) |
| Caregiver previously sought care for their child’s current illness at least once | | 9 | | (28.1) |
| Previous time seeking care resulted in hospital admission and subsequent discharge (N=9) | | 3 | | (33.3) |
| Assessment to Admission (N=32) | |  | |  |
| Time duration (hours) between patient assessment and admission, Median (IQR) | | 1.9 | | (0.9 – 3.1) |
| Admission decision made by: | |  | |  |
| Medical Officer | | 27 | | (78.1) |
| Intern Doctor | | 5 | | (21.9) |
| Caregiver consulted on day of admission on what patient journey will look like | | 6 | | (18.8) |
| Topics discussed with caregiver during admission consultation (N=6) | |  | |  |
| Suspected cause of patient’s illness | | 3 | | (50.0) |
| Hospital care plan | | 2 | | (33.1) |
| Types of barriers they may face | | 1 | | (16.7) |
| Estimated length of hospital stay | | 1 | | (16.7) |
| What recovery will look like | | 0 | | (0) |
| Admission to Discharge Order (N=31) | |  |  | |
| Time duration (days) between admission and discharge order, Median (IQR) | | 2.9 | | (1.9 – 4.8) |
| Discharge planning observed among care team | 22 | | | (71.0) |
| Time duration (days) between discharge planning and discharge, Median (IQR) (N=22) | 0 | | | (0 – 2) |
| Discharge planning led by: (N=22) |  | | |  |
| Medical Officer | 13 | | | (59.1) |
| Pediatrician | 5 | | | (22.7) |
| Intern Doctor | 4 | | | (18.2) |
| Caregiver was consulted during discharge planning (N=22) | 8 | | | (36.4) |
| Discharge Order to Discharge (N=31) |  | | |  |
| Time duration (hours) between discharge order and discharge, Median (IQR) | 3.0 | | | (2.3 – 6.4) |
| Discharge ordered by: |  | | |  |
| Medical Officer | 19 | | | (61.3) |
| Pediatrician | 6 | | | (19.4) |
| Intern Doctor | 6 | | | (19.4) |
| Patient was referred for post-discharge follow-up | 3 | | | (9.68) |
| Caregiver consulted on referral date & location (N=3) | 1 | | | (33.3) |
| Referral process barriers (N=3) |  | | |  |
| Language barriers between patient/caregiver and health workers | 0 | | | 0 |
| Lapse in patient/caregiver-provider communication | 1 | | | (33.33) |
| Lapse in provider-provider communication | 1 | | | (33.33) |
| Stock out of referral forms | 0 | | | 0 |
| Absence or unavailability of caregiver | 0 | | | 0 |
| Caregiver was educated on post-discharge care | 7 | | | (22.6) |
| Discharge education provided by: (N=7) |  | | |  |
| Medical Officer | 1 | | | (14.2) |
| Intern Doctor | 3 | | | (42.9) |
| Nurse | 3 | | | (42.9) |
| Discharge Education Topics Discussed (N=7) |  | | |  |
| Nutrition | 5 | | | (71.4) |
| Hygiene | 4 | | | (57.1) |
| Medication type/purpose | 3 | | | (42.9) |
| Medication management | 3 | | | (42.9) |
| Immunization | 3 | | | (42.9) |
| Mosquito net use | 1 | | | (14.3) |
| Medication side effects | 1 | | | (14.3) |
| Recovery | 1 | | | (14.3) |
| Warning signs | 1 | | | (14.3) |
| What to do if warning signs appear | 1 | | | (14.3) |
| Caregiver asked questions during discharge education process (N=7) | 2 | | | (28.6) |
| Discharge education barriers (N=7) |  | | |  |
| Language barriers between patient/caregiver and health workers | 2 | | | (28.57) |
| Lapse in patient/caregiver-provider communication | 1 | | | (14.29) |
| Lapse in provider-provider communication | 1 | | | (14.29) |
| Stock out of patient education materials | 1 | | | (16.67)* |
| Stock out of discharge forms | 0 | | | 0 |
| Absence or unavailability of caregiver | 0 | | | 0 |
| *missing=1 |  | | |  |

| **S4 Table B. Caregiver Satisfaction** |  |  |
| --- | --- | --- |
|  | **Total (N= 30)** | |
|  | n | % |
| Quality of discharge consultation |  |  |
| Very good | 7 | 23.33 |
| Good | 15 | 50.00 |
| Fair | 7 | 23.33 |
| Not sure | 1 | 3.33 |
| Health worker spent enough time preparing caregiver for discharge |  |  |
| Yes, definitely | 2 | 6.67 |
| Yes, to some extent | 23 | 76.67 |
| No, not really | 3 | 10.00 |
| No, definitely not | 1 | 3.33 |
| Not sure | 1 | 3.33 |
| Health worker explained things in a way that was easy to understand |  |  |
| Yes, definitely | 3 | 10.00 |
| Yes, to some extent | 26 | 86.67 |
| No, not really | 1 | 3.33 |
| No, definitely not |  |  |
| Not sure |  |  |
| Health worker gave caregiver an opportunity to ask questions or raise concerns about their child's discharge |  |  |
| Yes, definitely | 2 | 6.67 |
| Yes, to some extent | 13 | 43.33 |
| No, not really | 12 | 40.00 |
| No, definitely not | 2 | 6.67 |
| Not sure | 1 | 3.33 |
| Health worker involve caregiver as much as they wanted to be in decisions about their child's discharge |  |  |
| Yes, definitely | 1 | 3.33 |
| Yes, to some extent | 17 | 56.67 |
| No, not really | 10 | 33.33 |
| No, definitely not | 2 | 6.67 |
