# Supplementary material for: Transitions from hospital to home: A mixed methods study to evaluate pediatric discharges in Uganda
Source: PLOS Glob Public Health. 2023 Sep 13;3(9):e0002173. doi: 10.1371/journal.pgph.0002173 (PMC10499195; doi:10.1371/journal.pgph.0002173)
Supplement: Attachment — Submitted filename: RESPONSE TO REVIEWERS.docx [file pgph.0002173.s006.docx]

**Date: June 19, 2023**

Dear Editor and Reviewers,

Please find enclosed our revised manuscript entitled “Transitions from hospital to home: A mixed methods study to evaluate pediatric discharges in Uganda” for consideration for publication in PLOS Global Health.

We thank the PLOS Global Health Editorial Board and the reviewers for the thoughtful comments and suggestions. Enclosed are the responses and revisions to the comments. There is no doubt that these changes have increased the quality of the manuscript and we look forward to your deliberation.

Thank you for your consideration.

Sincerely,

Dr. Matthew O. Wiens, PhD; PharmD

Assistant Professor

Department of Anesthesiology, Pharmacology & Therapeutics | Institute for Global Health

The University of British Columbia | BC Children's Hospital

938 W 28th Ave | Vancouver BC | V5Z 4H4 Canada

Phone: 604 997 7753; Email: mowiens@outlook.com

**RESPONSE TO REVIEWERS**

Manuscript reference number: PLOS GLOBAL PUBLIC HEALTH PGPH-D-23-00725

Title: Transitions from hospital to home: A mixed methods study to evaluate pediatric discharges in Uganda.

**Journal Requirements:**

**Comment:**1. Please include a complete copy of PLOS’ questionnaire on inclusivity in global research in your revised manuscript. Our policy for research in this area aims to improve transparency in the reporting of research performed outside of researchers’ own country or community. The policy applies to researchers who have travelled to a different country to conduct research, research with Indigenous populations or their lands, and research on cultural artefacts. The questionnaire can also be requested at the journal’s discretion for any other submissions, even if these conditions are not met. Please find more information on the policy and a link to download a blank copy of the questionnaire here: https://journals.plos.org/globalpublichealth/s/best-practices-in-research-reporting. Please upload a completed version of your questionnaire as Supporting Information when you resubmit your manuscript.

**Response:** This is now included.

**Comment:** 2. Please provide separate figure files in .tif or .eps format.

For more information about figure files please see our guidelines: LINK

https://journals.plos.org/globalpublichealth/s/figures

https://journals.plos.org/globalpublichealth/s/figures#loc-file-requirements

**Response:** Supplementary figures are now provided in separate .tif files.

**Comment:** Please review your reference list to ensure that it is complete and correct. If you have cited papers that have been retracted, please include the rationale for doing so in the manuscript text, or remove these references and replace them with relevant current references. Any changes to the reference list should be mentioned in the rebuttal letter that accompanies your revised manuscript. If you need to cite a retracted article, indicate the article’s retracted status in the References list and also include a citation and full reference for the retraction notice.

**Response:** We have carefully reviewed our reference list to ensure completeness and correctness. We’ve since added the following references, which were used when addressing reviewer comments.

10.	World Health Organization. The Global Health Observatory: Skilled health professionals density (per 10 000 population). Accessed June 19, 2023. <https://www.who.int/data/gho/data/indicators/indicator-details/GHO/skilled-health-professionals-density-(per-10-000-population)>

11.	Wemon and ACHEST. Uganda’s Human Resources for Health: Paradoxes and Dilemmas.; 2019. Accessed June 19, 2023. https://www.wemos.nl/wp-content/uploads/2019/11/Wemos_Country-report-Uganda-2019_Ugandas-Human-resources-for-health_Paradoxes-and-dilemmas.pdf

13.	The World Bank. Uganda | Data. Accessed June 19, 2023. https://data.worldbank.org/country/uganda

14.	Wiens M, Trawin J, Komugisha C, et al. Smart Discharges Transition to Scale. Published online 2023. doi:doi:10.5683/SP3/IDLGNN

22.	Muraya K, Ogutu M, Mwadhi M, et al. Applying a gender lens to understand pathways through care for acutely ill young children in Kenyan urban informal settlements. Int J Equity Health. 2021;20(1):17. doi:10.1186/s12939-020-01349-3

23.	Zakayo SM, Njeru RW, Sanga G, et al. Vulnerability and agency across treatment-seeking journeys for acutely ill children: how family members navigate complex healthcare before, during and after hospitalization in a rural Kenyan setting. Int J Equity Health.

**REVIEWER # 1**

**Comment:** This is an important topic of emerging interest to the global public health community. The objectives and study design are clear and the manuscript well-written. A few areas would benefit from additional information:

**Response**: Thank you for your careful review of our manuscript and for these encouraging comments. We have responded to each of your specific points below.

**Comment:** I would like to have seen some more detail in Methods following "Study nurses, providing 12 hours per day... ". For example, how did they witness the admission process, family expectations and understanding of the treatment plan (which may extend from during admission to after discharge) and communication between health staff and families?

**Response:** Thank you for this comment. We have added more detail to the methods section to explain how the study nurses witnessed the process.

Only children admitted to the hospital during the daily observational hours were enrolled. Therefore, we updated the Phase I; Patient Journey Mapping eligibly criteria to state:

“We aimed to enroll a total of 32 children under 5-years of age who were admitted to participating facilities with a proven or suspected infection during the 12 hour per day, 7 days per week observation period.”

Phase 1 study procedures section now reads:

“The study nurses physically observed any interactions between the healthcare provider and the caregiver and documented what they saw and heard using a survey comprised of a series of checklists and close-ended questions with some open-entry questions^10^. The main aspects observed included; 1) description of the admission process and caregiver involvement in admission decisions; 2) communication between the healthcare providers and caregivers the child’s diagnosis, the cause of the illness, estimated length of stay, hospital care plan and the likely barriers to stop caregivers from staying in the hospital; 3) the discharge planning process, discharge education, discharge orders, referral and follow-up appointments. The study nurses further identified and documented the barriers at each of these care points. At the start of each 12 hour observation period, study nurses briefly interviewed the night shift healthcare team to obtain any details relevant to the enrolled patient’s journey that occurred during the non-observation period. Telephone interviews were conducted with the child’s primary caregiver 72 hours after discharge to obtain post-discharge process outcomes and patient experience measures.”

We also added this as a fourth study limitation stating:

“Fourth, we did not observe the patients journey 24 hours per day and thus may have missed documenting some relevant events. However, study nurses were able to observe the discharge process for all enrolled patients as discharges occurred during daytime hours.”

**Comment:** In results, at line 212, financial constraints to admission leading to delays, and/or seeking local care alternatives are well described in the literature - were these investigated? If, so it would be helpful to report on these.

**Response:** We agree that the barriers described above are important. Indeed we have previously explored some of these in our prior work, especially as it related to seeking care during the post-discharge period. See: Krepiakevich et a. Out of pocket costs and time/productivity losses for pediatric sepsis in Uganda: a mixed-methods study. *BMC Health Serv Res*. 2021;21(1):1-9. doi:10.1186/s12913-021-07272-9 and Nemetchek et al. Exploring healthcare providers’ perspectives of the paediatric discharge process in Uganda: a qualitative exploratory study. *BMJ Open*. 2019;9(9):e029526. doi:10.1136/bmjopen-2019-029526. Although we did not explore barriers that occurred prior to the initial admission for this present study, we did capture barriers experienced during the hospital to home transition as well as during the post-discharge follow-up referral process.

**Comment:** I thought the reporting of '...two were informed about their child’s hospital care plan, one was informed about the types of barriers they may face during treatment.' was very helpful - more details on how this was sought (systematically?) would add value.

**Response:** We agree that more details would add value and apologize for that oversight. We’ve updated the methods section to better describe our data collection tools and process. Please also note that we have included a reference (14) to our data collection tools that are now available on Borealis, the Canadian Dataverse Repository.

Phase I Study Procedures section now states the following:

“The study nurses physically observed any interactions between the healthcare provider and the caregiver and documented what they saw and heard using a survey comprised of a series of checklists and close-ended questions with some open-entry questions^14^. The main aspects observed included; 1) description of the admission process and caregiver involvement in admission decisions; 2) communication between the healthcare providers and caregivers the child’s diagnosis, the cause of the illness, estimated length of stay, hospital care plan and the likely barriers to stop caregivers from staying in the hospital; 3) the discharge planning process, discharge education, discharge orders, referral and follow-up appointments. The study nurses further identified and documented the barriers at each of these care points. At the start of each 12 hour observation period, study nurses briefly interviewed the night shift healthcare team to obtain any details relevant to the enrolled patient’s journey that occurred during the non-observation period.”

**Comment:** Another previously reported issue is problematic staff-mother communication with mothers sometimes feeling looked down on and blamed - was this sought or identified?

**Response:** You are right, and this is a very important issue in health worker-caregiver interactions. Unfortunately, in this study we did not capture the “tone” of the interactions (either perceived or observed). This is something we feel we should address in future work.

**Comment:** Under 'discharge planning' in the Results, when did discharge planning begin?

**Response:** We have provided more information on when discharge planning begins. It now reads: “Discharge planning among the patient’s care team was observed in 71% of cases (n=22), though planning typically began on the same day of discharge (55%, n=12) and sometimes (42%, n=5) in less than 4 hours prior to discharge.”

**Comment:** Under 'discharge education' how many mothers/carers asked questions about what was being recommended post-discharge?

**Response:** We apologize for this oversight. We have updated Supplementary Table 1 as well as the text to state: “During the education process, two caregivers (25%) asked questions pertaining to cost/billing, their child’s diagnosis, and/or discharge timelines.”

**REVIEWER # 2**

**Comment:** In methods section Lines 105 - 106. It is stated that the study had 3 phases: journey mapping, discharge process mapping and Focus Group discussions (FGDs). The first 2 Phases are self-explanatory. Can the authors describe more the FGDs phase?

**Response:** We updated the text to read: “FGDs were conducted with primary caregivers and fathers of discharged patients to explore caregiver perspectives of barriers and solutions to effective hospital discharge.”

**Comment:** Still in the methods section Lines 173-176. All FGDs were digitally recorded, transcribed into English. Was translation not done?

**Response:** We apologize for skipping this important aspect. We have corrected this in the manuscript and now reads: “All FGDs were transcribed verbatim and translated into English by external individuals fluent in the languages with no previous interactions with study participants.”

**Comment:** Table 2: Harmonize figures in the table and text (length of hospital stay, days, [Median IQR]).

**Response:** Thank you very much for catching that typo. This has been corrected.

**Comment:** Figure1: Pediatric Discharge Process Map. The first 2 child exit points do not appear to be discharges as the children were managed as outpatients.

**Response:** Thank you for catching this error. It has now been corrected to state “Child sent home”

**Comment:** Correct spelling of "cue" to "queue" in box with - CG did not purchase medication for other reasons (money, long cue etc)

**Response:** Thank you. We have corrected the spelling

**REVIEWER # 3:**

**Comment:** This is a clear straightforward paper making important points based on a careful multi-method study with limitations clearly presented. I have only very minor comments:

**Response:** Thank you for this encouraging feedback.

**Comment:** In the abstract in the first para, there is a key sentence stating '...planning in many hospitals leading to poor implementation'. I suggest that the word 'leading' is replaced with 'contributing'. Although a very minor comment, the sentence highlights the inadequate attention in the paper to the range of structural/organisational drivers of poor post-discharge planning and patient outcomes, and therefore to how complex it can be to introduce and sustain change. Relatedly, a sentence or two highlighting this challenge and complexity could be added to the discussion, where there are many potential improvements listed without acknowledgement of the challenges of implementing such interventions beyond the context of trials.

**Response:** We have replaced the word leading with “contributing”. This is how the sentence reads; “However, insufficient attention has been paid to post discharge planning in many hospitals contributing to poor implementation”.

We’ve also added another sentence to the second paragraph in the Discussion section to state: “Although the reasons for this are complex, managing multiple objectives and competing health systems demands are main barriers to both program implementation and sustainability.”

**Comment:** in the introduction para 1, is the lack of awareness about post-discharge vulnerability a lack of awareness among policy makers, providers or parents (or all)

**Response:** We have clarified that lack of awareness of post-discharge vulnerability amongst both healthcare providers and caregivers.

**Comment:** in the introduction it would be good to know a little more about the hospitals, including staffing levels in relevant wards and cost policies of admission

**Response:** Thank you for your useful suggestion. We felt that the suggested information may fit better in the Design & Setting section. Although we were unable to find reliable information for each of our study sites, we updated the methods section to provide an overview of staffing levels and healthcare costs within the Ugandan health system. This section now reads:

Uganda’s skilled health professionals density of 7.42 per 10,000 population remains well below the WHO recommended of 23 per 10,000 population, with highest staffing shortages experienced among health centers, public facilities, and those in rural areas. Healthcare services are free of charge at government owned health facilities and vary in premium fees at private-not-for-profit (PNFP) health facilities; however, patients incur substantial economic burden in accessing care regardless of facility ownership. A 2021 mixed methods study on out-of-pocket patient costs in Uganda found that total hospitalization costs, excluding missed wages, ranged from 62.1 USD among public sector regional referral hospitals to 124.5 USD among PNFP hospitals, while approximately 42% of the population lives on less than $1.15 USD per day.

**Comment:** methods - the process maps across the very different hospitals were brought together into one - why was this done; would it not have been helpful to keep them separate, and look for similarities and differences (and reasons for difference?); how much could/should post-discharge differ by patient group - for example those admitted with an acute illness but also suffering from HIV, epilepsy, malnutrition

**Response:** We agree that looking at the process maps individually is very valuable. However our main purpose in this was to define the most common elements in this process to be able to work towards a process improvement which could be integrated across different types of sites. This was one main motivation for choosing the sites we did – variability in ownership (two types of private not for profit and 1 public site). We have provided the individual-level process maps in the Supplementary File 2 (Suppl. Figures 2, 3, and 4) as we feel these are important, but do not fit into the main list of figures allowable in the main manuscript. We did not explore differences by patient group but agree that this is an import aspect to explore in future work.

**Comment:** findings/discussion - it may be that there are some interesting differences in some of the gender and other dynamics across the different Ugandan settings, and with other nearby countries? See for example Muraya K, Ogutu M, Mwadhi M, Mikusa J, Okinyi M, Magawi C, et al. Applying a gender lens to understand pathways through care for acutely ill young children in Kenyan urban informal settlements. Int J Equity Health. 2021;20(1):17. and Zakayo SM, Njeru RW, Sanga G, Kimani MN, Charo A, Muraya K, et al. Vulnerability and agency across treatment-seeking journeys for acutely ill children: how family members navigate complex healthcare before, during and after hospitalisation in a rural Kenyan setting. Int J Equity Health. 2020;19(1):136.

**Response:** Thank you for providing these resources. We have incorporated these findings into our discussion section which now reads: “Involving both male and female caregivers in discharge planning is important as gender roles, which can vary by culture and geographical region, can affect decision-making processes for childhood illness.”

**Comment:** ethics section in methods - exercise should be plural?

**Response:** The word exercise has been changed to plural to read as exercises in the methods section

**Comment:** throughout, data should be plural? (currently inconsistent)

**Response:** Thank you for catching this inconsistency. We have replaced wording so that data is pluralized throughout the document
